# Study on Dicyandiamide-Imprinted Polymers with Computer-Aided Design

**DOI:** 10.3390/ijms17111750

**Published:** 2016-10-26

**Authors:** Dadong Liang, Yan Wang, Songyang Li, Yuqing Li, Miliang Zhang, Yang Li, Weishuai Tian, Junbo Liu, Shanshan Tang, Bo Li, Ruifa Jin

**Affiliations:** 1 College of Resources and Environment, Jilin Agricultural University, Changchun 130118, China; liangdadong@aliyun.com (D.L.); lsy3153204@126.com (S.L.); liyuqingstudent@163.com (Y.L.); m18843186104@163.com (M.Z.); z1119517228@163.com (Y.L.); tian835116164@163.com (W.T.); 2 Department of resources engineering, Guangxi Modern Polytechnic College, Guangxi 547000, China; m15672869883@163.com; 3 Hebei Provincial Key Laboratory of Inorganic Nonmetallic Materials, College of Materials Science and Engineering, North China University of Science and Technology, Tangshan 063009, China; 4 College of Chemistry and Chemical Engineering, Chifeng University, Chifeng 024000, China; ruifajin@163.com

**Keywords:** dicyandiamide, molecular imprinting, molecularly imprinted polymer, computer simulation

## Abstract

With the aid of theoretical calculations, a series of molecularly imprinted polymers (MIPs) were designed and prepared for the recognition of dicyandiamide (DCD) via precipitation polymerization using acetonitrile as the solvent at 333 K. On the basis of the long-range correction method of M062X/6-31G(d,p), we simulated the bonding sites, bonding situations, binding energies, imprinted molar ratios, and the mechanisms of interaction between DCD and the functional monomers. Among acrylamide (AM), *N*,*N*’-methylenebisacrylamide (MBA), itaconic acid (IA), and methacrylic acid (MAA), MAA was confirmed as the best functional monomer, because the strongest interaction (the maximum number of hydrogen bonds and the lowest binding energy) occurs between DCD and MAA, when the optimal molar ratios for DCD to the functional monomers were used, respectively. Additionally, pentaerythritol triacrylate (PETA) was confirmed to be the best cross-linker among divinylbenzene (DVB), ethylene glycol dimethacrylate (EGDMA), trimethylolpropane trimethylacrylate (TRIM), and PETA. This is due to the facts that the weakest interaction (the highest binding energy) occurs between PETA and DCD, and the strongest interaction (the lowest binding energy) occurs between PETA and MAA. Depending on the results of theoretical calculations, a series of MIPs were prepared. Among them, the ones prepared using DCD, MAA, and PETA as the template, the functional monomer, and the cross-linker, respectively, exhibited the highest adsorption capacity for DCD. The apparent maximum absorption quantity of DCD on the MIP was 17.45 mg/g.

## 1. Introduction

Molecular imprinting is a class of highly efficient molecular recognition technologies. Targeted molecules with suitable size, shape, and functional group can be selectively absorbed by the selective recognition sites of the molecularly imprinted polymers (MIPs) [1,2]. To prepare the MIPs, templates and functional monomers should be employed. The functional monomer molecules are allowed to self-assemble around the template molecules to form the template-monomer complexes. Then, cross-linkers are added, allowing the crosslinking polymerization reactions to be performed around the complexes. Finally, the template molecules are extracted, and the MIPs with tailor-made recognition sites are obtained. So far, due to the advantages of MIPs, namely excellent molecular recognition performance, low cost, high physical and chemical stability, and application universality, they have received much attention and become attractive in many fields, such as solid phase extraction [3,4], chiral separation [5], immune-like assay [6], antibody simulation [7], chemical sensors [8,9], drug delivery [10], proteomics [11], and wastewater treatment [12].

In recent years, many scientific groups have studied MIPs with the aid of theoretical calculations. Del Sole et al. reported the prepolymerization interaction between nicotinamide and methacrylic acid in different solvents and the computational investigation on the complex to obtain a better understanding of hydrogen-bonding interactions [13]. Pietrzyk et al. simulated the MIP-melamine complex by using the density functional theory (DFT) [14]. Liu et al. designed and prepared magnetic imprinted polymers [15]. Wei et al. investigated the mechanism of 17β-estradiol imprinting with dynamic simulations to select the most suitable monomers [16]. Yañez et al. provided a computation to screen commonly used monomers and select the most suitable monomers for synthesizing cholate-imprinted and non-imprinted polymer [17]. In sum, the theoretical calculations may reduce the time required for experimental studies. More importantly, the molecular imprinting mechanism, such as the interactions between the monomer molecules and the template molecules, may be analyzed and explained.

There are some key factors, such as the functional monomer, imprinted molar ratio, and cross-linker, which significantly affect the adsorption properties of the MIPs. Therefore, in order to obtain a MIP with high performances, functional monomer, imprinted molar ratio, and cross-linker should be chosen carefully. For selecting appropriate functional monomers, the strengths of interactions between functional monomers and templates should be evaluated. Usually, more interaction sites and lower values of the binding energies should result in stronger interactions. Thus, the number of interaction sites and the values of binding energies between the templates and the functional monomers may be used as the criteria for choosing the functional monomers and the imprinted molar ratios. Moreover, the choice of cross-linker is also of vital importance. The strengths of the interactions between the templates and the cross-linkers should be weak, and that between the functional monomers and the cross-linkers should be strong. Otherwise, the cross-linkers may compete with the monomers, which may result in poor recognition properties of the MIPs for the targeted molecules. Therefore, to choose an appropriate cross-linker, the binding energies between the cross-linkers and the templates/functional monomers should be calculated.

**Figure 1:**
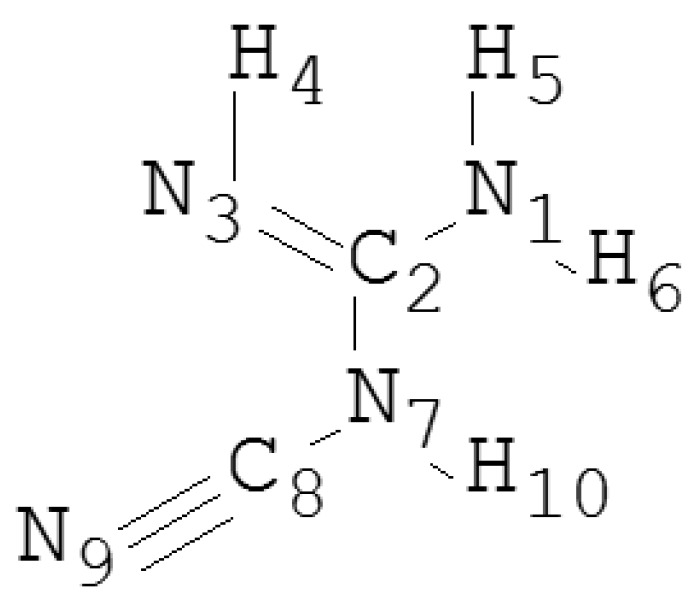
Structure of DCD.

In 2013, it was reported that dicyandiamide (DCD, see Figure 1), which is toxic to human beings and especially to infants and children, had been detected in the milk produced in New Zealand. One reason for the utilization of DCD in agriculture is that DCD may decrease nitrate leaching and nitrous oxide emissions in irrigated land [18,19]. Recently, more and more researchers have devoted their efforts to developing the molecular imprinting technology for recognizing DCD. For example, Liu et al. have synthesized MIPs by involvement of both DCD and melamine as co-templates [20]. Liu et al. have developed a turn-off optosensing approach for DCD detection in milk samples on the basis of quantum dots labeled on the surface of MIP [21]. However, to the best of our knowledge, there is no report on design and preparation of the MIPs with specific adsorption properties for DCD, with the aid of theoretical calculations.

In this manuscript, with the aim of obtaining MIPs with the property of selectively adsorbing DCD, theoretical calculations were carried out to provide key information for design and preparation of the MIPs. Herein, DCD was used as the template. Acrylamide (AM), methacrylic acid (MAA), *N*,*N*’-methylenebisacrylamide (MBA), and itaconic acid (IA) were considered as the functional monomers. Divinylbenzene (DVB), ethylene glycol dimethacrylate (EGDMA), trimethylolpropane trimethylacrylate (TRIM), and pentaerythritol triacrylate (PETA) were regarded as the candidates of the cross-linkers. The DFT with long range correction method was used for calculating the bonding sites, bonding situations, binding energies, and the mechanism of the interactions between DCD and the functional monomers. Based on the calculated results, the appropriate functional monomer, imprinted molar ratio, and cross-linker were confirmed. Then, a series of MIPs were prepared via the precipitation polymerization using acetonitrile as the dispersion solvent at 333 K. The adsorption capacities of DCD on the MIPs were also investigated.

## 2. Results and Discussion

### 2.1. Selection of the Appropriate Calculated Method

DFT calculations have been carried out in order to study the DCD imprinted polymers. There are a lot of DFT methods, but different methods should be applied in different research systems. The choice of calculated methods has a great influence on the accuracy of the calculated results. Herein, we simulated the structural parameters of DCD using the B3LYP, PBE1PBE, wB97XD, M062X, and LC-wPBE methods with the 6-31G(d,p) basis set. These calculation methods have been used in the previous researches on the MIPs. For example, Khan et al. designed a new computational model capable of understanding the nature of interaction between the template and the functional monomers by employing the B3LYP method [22]. Diñeiro et al. used the B3LYP method to design the MIPs for voltammetric sensing of homovanillic acid [23]. Piacham et al. investigated the molecularly imprinted nanospheres for selective recognition of α-tocopherol succinate by using the B3LYP method [24]. Liu et al. used the quantum chemical method PBE1PBE to investigate the interaction between melamine and acrylamide in the MIPs [25]. Otherwise, wB97XD [26,27], M062X [28,29], and LC-wPBE [30] methods have also been used for simulating the MIPs.

Herein, the appropriate calculated method was confirmed through comparing the calculated results and the experimental data [31]. As shown in Table 1, the computational structural parameters and the experimental data are highly similar, which indicates that the DFT is effective and reliable in the optimization of the geometry structures. Among these methods, the M062X/6-31G(d,p) method should be the most appropriate calculated method, because the structural parameters calculated on the basis of the M062X method are the closest values to the experimental data. For example, the practical bond length of N9-C8 is 1.22 Å, and the value given by the M062X method is 1.18 Å. In contrast, the values given by the wB97XD, PBE1PBE, and LC-wPBE methods are all 1.16 Å, and the one from the B3LYP method is 1.17 Å. Moreover, the practical bond length of N1-C2 is 1.34 Å. The value given by the M062X method is 1.28 Å, while the values given by other methods are all 1.27 Å. As a result, the M062X/6-31G(d,p) method was selected as the appropriate calculated method.

### 2.2. Forecast of the Active Sites for the Template and Function Monomers

**Figure 2:**
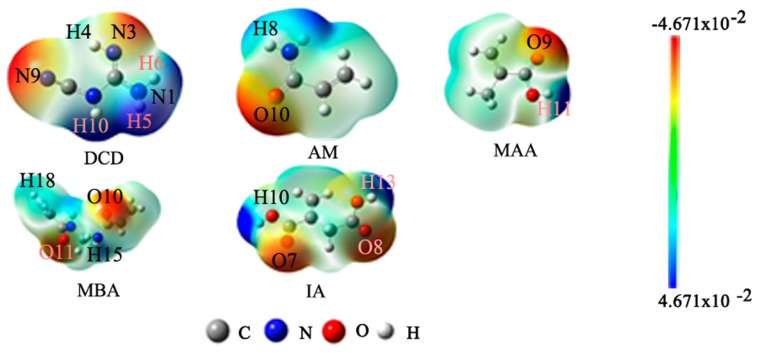
MEP populations of DCD, AM, MAA, MBA, and IA.

DCD was used as the template; AM, MAA, MBA, and IA were considered as the functional monomers. In order to predict the electrophilic and nucleophilic reactive sites of the template and the functional monomers, the molecular electrostatic potentials (MEPs) of DCD, AM, MAA, MBA, and IA were studied by using the M062X/6-31G(d,p) method. As shown in Figure 2, the atoms H4, H5, H6, and H10 of DCD exhibit significant positive charges, which reveal that they are proton donors. The atoms N3 and N9 of DCD own obviously negative charges, which indicate that they are proton acceptors. On the basis of the same analyses, it can be concluded that, in AM, the atom H8 is the proton donor, and the atom O10 is the proton acceptor. In MAA, the atom H11 is the proton donor, and the atom O9 is the proton acceptor. In MBA, the atoms H15 and H18 are proton donors, and the atoms O10 and O11 are proton acceptors. In IA, the atoms H10 and H13 are proton donors, and the atoms O7 and O8 are proton acceptors.

### 2.3. Selection of Functional Monomer Based on Theoretical Calculations

**Figure 3:**
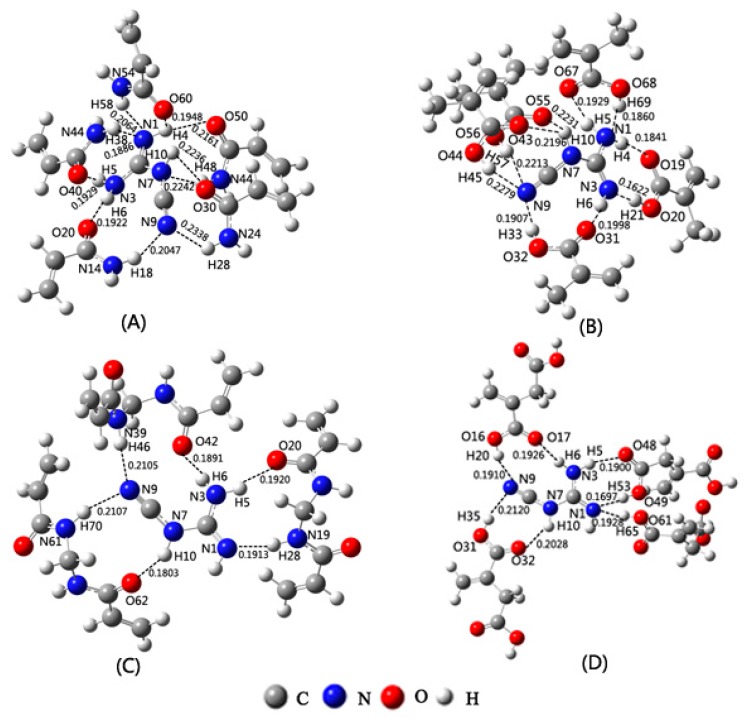
Models of complexes formed from DCD and the functional monomers of AM (**A**); MAA (**B**); MBA (**C**); and IA (**D**).

AM, MAA, MBA, and IA have been considered as the functional monomers. As shown in Figure 3, the models of the complexes constructed by DCD and the functional monomers (AM, MAA, MBA, and IA) were calculated, respectively. The calculated results indicate that the optimal molar ratios for DCD to AM, MAA, MBA, and IA are 1:5, 1:5, 1:3, and 1:4, respectively. Applying these imprinting molar ratios, the complexes exhibited the lowest energies and the maximum number of bonds between DCD and the functional monomers, respectively. The numbers of bonds between DCD and AM, MAA, MBA, and IA are respectively 10, 10, 6, and 7 in the complexes. The lengths of these bonds are all within the range of H-bond length (0.1622~0.2279 nm) [22]. Additionally, the roles of the reactive sites of DCD and the functional monomers are consistent with the results of the MEP analysis. For example (see Figure 3B), when MAA acted as the functional monomer, the proton donors (H4, H5, H6, and H10) of DCD form the H-bonds with the proton acceptors (O19, O67, O31, and O43 (O55)) of MAA, respectively. Moreover, the N3 and N9 atoms (proton acceptors) of DCD form the H-bonds with the H21 and H33 (H45, H57) atoms (proton donors) of MAA, respectively. The values of binding energies of DCD and different functional monomers (Δ*E*_B1_) were listed in Table 2. As shown in Table 2, the values of Δ*E*_B1_ are in the order of Δ*E*_B1_(DCD-MAA) < Δ*E*_B1_(DCD-AM) < Δ*E*_B1_(DCD-IA) < Δ*E*_B1_(DCD-MBA), which may indicate that the interaction between DCD and MAA has the highest strength among the complexes formed from DCD and the functional monomers. As a result, among these functional monomers, MAA should be the best one for the preparation of the MIPs with the properties of recognizing DCD, and the best imprinting molar ratio should be 1:5.

As shown in Figure 3B, one DCD molecule is surrounded by five MAA molecules. There are a large number of interactions between the DCD molecule and the MAA molecules. For example, the H4, H5, H6, and H10 atoms of the DCD molecule interact with the O19, O67, O31, and O43 (O55) atoms (proton accepters) of the MAA molecules, respectively. The N1, N3, and N9 atoms (proton accepters) of the DCD molecule interact with the H69, H21, and H33 (H45, H57) atoms of the MAA molecules. From Figure 3B, it could be found that there is no self-association of the MAA functional monomer in the concentration range studied in the work. This is in agreement with the conclusion that the self-association might not appear in the low concentration of the functional monomer [32].

### 2.4. Selection of Cross-Linker Based on Theoretical Calculations

The molecules DVB, EGDMA, TRIM, and PETA were commonly used as the cross-linkers in the preparation of the MIPs, thus, herein they were selected as the cross-linkers in our study. The binding energies (Δ*E*_B2_) between DCD/MAA and cross-linkers were calculated at the M062X/6-31G(d,p) level. As shown in Table 3, the Δ*E*_B2_ values between DCD and different cross-linkers are in the increase order of Δ*E*_B2_(DCD-TRIM) < Δ*E*_B2_(DCD-EGDMA) < Δ*E*_B2_(DCD-DVB) < Δ*E*_B2_(DCD-PETA). The Δ*E*_B2_ values between MAA and different cross-linkers are in the decrease order of Δ*E*_B2_(MAA-DVB) > Δ*E*_B2_(MAA-EGDMA) > Δ*E*_B2_(MAA-TRIM) > Δ*E*_B2_(MAA-PETA). This shows that the Δ*E*_B2_ value (−33.61 kJ/mol) between DCD and PETA is the highest, and the Δ*E*_B2_ value (−43.58 kJ/mol) between MAA and PETA is the lowest. That is to say, the weakest interaction occurs between PETA and DCD, and the strongest interaction occurs between PETA and MAA. Under the rule that the strength of interaction between the template and cross-linker should be weak and that between the functional monomer and cross-linker should be strong, PETA was chosen as the best cross-linker for the DCD-MIPs preparation among these cross-linkers.

### 2.5. Adsorption Property of the MIPs

To obtain a MIP with good adsorption capacity for DCD, a series of MIPs and non-imprinted polymers (NIPs) were prepared using different functional monomers, imprinting molar ratios, and cross-linkers. The adsorption experiments were designed and performed on the basis of the results of theoretical calculations.

**Figure 4:**
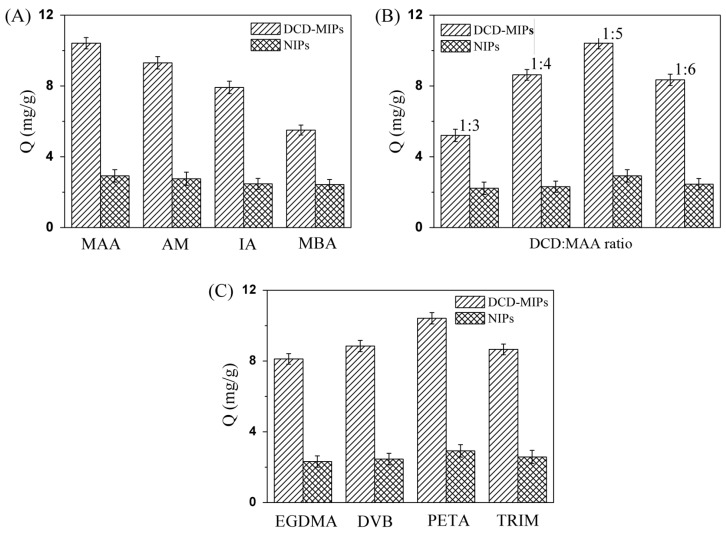
Adsorption capacities of MIPs and NIPs with different functional monomers (**A**); different imprinting ratios (**B**); and different cross-linking agents (**C**). Adsorption conditions: MIPs or NIPs, 20.0 mg; DCD methanol solution, 100 mg/L, 10.00 mL; 4 h.

First of all, MIPs were prepared using different functional monomers (MAA, AM, IA, and MBA), when the best imprinting molar ratios of their own were applied and PETA was used as the cross-linker. As shown in Figure 4A, the MIPs with different functional monomers exhibit different adsorption capacities. The MIP, with MAA as the functional monomer, shows the highest adsorption capacity. The sequence for different functional monomers is MAA > AM > IA > MBA, according to the adsorption quantities. Thus, the MAA is the best monomer in our studies for DCD-MIP preparation.

In order to confirm the best molar ratio for DCD to MAA, the MIPs with different imprinting ratios were prepared, when MAA was used as the functional monomer and PETA was used as the cross-linker. As shown in Figure 4B, the MIP, whose molar ratio of DCD to MAA is 1:5, shows the maximum adsorption quantity. The order of different molar ratios is 1:5 > 1:4 > 1:6 > 1:3, according to the adsorption quantities.

Then, the experiments for the choice of cross-linker were carried out. In Figure 4C, the MIP was prepared using PETA as the cross-linker with the maximum adsorption quantity, when MAA was the monomer and the molar ratio of DCD to MAA is 1:5. The sequence of cross-linkers is PETA > DVB > TRIM > EGDMA, according to the adsorption quantities, which may indicate that PETA is the best cross-linker in our study.

To fully evaluate the adsorption properties of the MIPs, a series of NIPs were prepared and used for adsorbing DCD, and then their adsorption capacities were compared with that of the MIPs. These NIPs were prepared through the same process as the preparations of MIPs, excepting the absence of the template (DCD). As shown in Figure 4A–C, the adsorption quantities of the MIPs are always significantly higher than that of the corresponding NIPs, which may be explained by the fact that the MIPs contain the imprinted cavities which match the DCD molecules.

**Figure 5:**
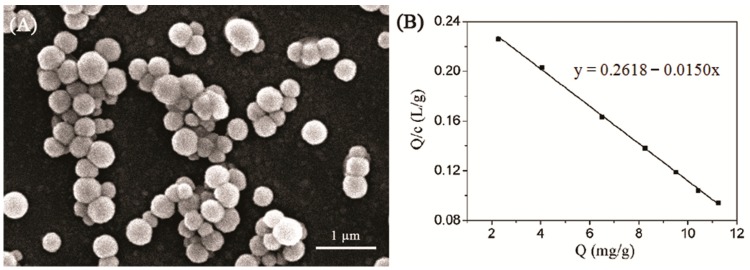
(**A**) The SEM of DCD-MIP with PETA as the cross-linker (imprinting molar ratio of DCD:MAA = 1:5); (**B**) The Scatchard plot of the DCD-MIP for DCD adsorption.

Finally, on the basis of above experimental results, a DCD-MIP was prepared using MAA as the functional monomer and PETA as the cross-linker, and the molar ratio of DCD to MAA is 1:5. The scanning electron microscope (SEM) photograph of the DCD-MIP is shown in Figure 5A. The average diameter of the MIP microspheres is about 300 nm. In order to obtain the information about the DCD binding characteristics of the DCD-MIP, the adsorption behavior of the DCD-MIP was investigated. The initial DCD concentrations were in the range of 10 to 120 mg/L. A Scatchard plot was used to evaluate the adsorption property of the DCD-MIP. As shown in Figure 5B, the Scatchard plot is a single straight line, which might indicate that the binding sites of the MIP toward DCD are equal in the studied concentration range. The linear regression equation is *Q*/*C* = 0.2618 − 0.0150*Q*. On the basis of this equation, it can be concluded that the *K*_d_ value and *Q*_max_ value of the DCD-MIP are 66.67 mg/L and 17.45 mg/g, respectively.

## 3. Materials and Methods

### 3.1. Chemicals and Reagents

AM, CYR, TRI, DCD, MAA, MAM, MBA, and IA were purchased from Aladdin Company (Shanghai, China). Acetic acid and methanol were obtained from Chemical Reagent Company (Beijing, China). DVB, EGDMA, PETA, TRIM, and 2,2-azobisisobutyronitrile (AIBN) were bought from Tianjin Guangfu Fine Chemical Research Institute (Tianjin, China).

### 3.2. Computational Methods

Initially, the structure of DCD (see Figure 1) was optimized using the Gaussian 09 software [33]. The PBE1PBE, M062X, LC-wPBE, wB97XD, and B3LYP methods and the 6-31G(d,p) basis set were carried out. Through comparing the calculated structure parameters with the experimental data, the M062X/6-31G(d,p) method was confirmed as the most reliable method. Utilizing this method, the structures of functional monomers, cross-linkers, and their pre-complexes were optimized. The conformations of DCD were also calculated, when it is surrounded by the monomers in acetonitrile. Moreover, the structures of different isomers of the complexes of DCD and the monomer with different imprinting molar ratios were optimized. The complexes with the lowest value of binding energy (ΔE_B_) and the maximum number of hydrogen bond were selected as the research objects. The basis set superposition error (BSSE) was used to correct the energies of the complexes [34]. The ΔE_B1_ of the complexes of the template and the monomer was calculated through Equation (1):

Δ*E*_B1_ = *E*_template–monomer_ − *E*_template_ − Σ*E*_monomer_ (1)

The Δ*E*_B2_ of the complexes of template (or monomer) and cross-linker was calculated by Equation (2):

Δ*E*_B2_ = *E*_template–cross-linker or monomer–cross-linker_ − *E*_template or monomer_ − *E*_cross-linker_ (2)

### 3.3. Preparation of MIPs and NIPs

In a typical process, the template (DCD, 33.6 mg, 0.400 mmol) and the functional monomer (MAA, 0.172 g, 2.00 mmol) were added in acetonitrile (40.00 mL) at room temperature and stirred for 24 h. Then, the cross-linker (PETA, 2.98 g, 10.0 mmol) and the initiator (AIBN, 20.0 mg, 0.122 mmol) were added. The solution was purged with nitrogen for 10 min to remove oxygen dissolved in the solvent. Then, it was sealed and incubated in a water bath at 333 K for 24 h.

The DCD molecules in the MIPs were extracted with methanol/acetic acid solutions (*v*/*v*, 8/2) in a soxhlet extractor. The extract procedures were repeated for several times until the DCD molecules could not be detected in the effluent. To remove acetic acid from the system, the methanol solution was loaded continuously for 12 h. Finally, the polymer microspheres were dried under vacuum at 333 K. As a reference, the NIP microspheres were prepared through the same processes excepting the absence of DCD.

### 3.4. Analysis of Adsorption Property

In a typical process, dry MIP or NIP microspheres (20.0 mg) were immersed in a DCD methanol solution (10.00 mL, 100 mg/L). The mixture was oscillated at room temperature for 4 h and then centrifuged. The supernatant was filtered using a membrane filter (0.22 μm). The concentration of solution was determined by monitoring the absorbance at the maximum absorbance wavelength of DCD (216 nm).

The equilibrium adsorption capacity (*Q*, mg/g) of MIPs for DCD was calculated according to Equation (3):

*Q* = (*C*_i_ − *C*_f_)*V*/*W*, (3)

where *C*_i_ and *C*_f_ (mg/L) are the initial and final concentrations of DCD, respectively. *V* (mL) is the volume of the solution. *W* (mg) is the weight of MIPs or NIPs.

The Scatchard equation is described via Equation (4):

*Q*/*C* = (*Q*_max_ − *Q*)/*K*_d_, (4)

where *Q* (mg/g) is defined as the DCD adsorption quantity of MIPs and *C* (mg/L) is the DCD concentration. *Q*_max_ (mg/g) and *K*_d_ (mg/L) are apparent maximum adsorption capacity and the dissociation constant, respectively.

## 4. Conclusions

In this manuscript, we prepared a series of MIPs via precipitation polymerization, using computer-aided design. Through comparing the calculated and experimental structure parameters of DCD, the M062X method was confirmed to be the appropriate calculation method, among the PBE1PBE, M062X, LC-wPBE, wB97XD, and B3LYP methods. Under this calculation method, MAA was confirmed as the appropriate functional monomer among AM, MAA, MBA, and IA; the best imprinting molar ratio of DCD to MAA is determined to be 1:5; and PETA was determined to be the best cross-linker among DVB, EGDMA, TRIM, and PETA. On the basis of the results of the theoretical calculations, a series of MIPs were prepared using different functional monomers, imprinting molar ratios, and cross-linkers. The results of the adsorption experiments indicate that the MIP exhibits the highest adsorption capacity for DCD when MAA is used as the functional monomer, PETA is used as the cross-linker, and the molar ratio for DCD to the MAA is 1:5. This conclusion is consistent with the results of theoretical calculations, which proves that the application of theoretical calculations in the field of molecular imprinting theory is effective and reliable. In sum, this work could contribute to the development of molecular imprinting technology and provide references for the selective separation, enrichment, and detection of DCD.

## Figures and Tables

**Table 1:** The calculated (at the wB97XD, B3LYP, PBE1PBE, LC-wPBE, and M062X levels with the 6-31G(d,p) basis set) and experimental structural parameters of DCD.

Species	wB97XD	B3LYP	PBE1PBE	LC-wPBE	M062X	Experimental
*R*(Å)						
N1–C2	1.27	1.27	1.27	1.27	1.28	1.34
N3–C2	1.37	1.37	1.36	1.36	1.37	1.37
N7–C2	1.42	1.42	1.42	1.42	1.42	1.36
N7–C8	1.33	1.33	1.33	1.33	1.33	1.28
N9–C8	1.16	1.17	1.16	1.16	1.18	1.22
*Φ*(°)						
N1–C2–N3	123	123	123	123	123	124
N1–C2–N7	121	121	121	121	121	120
N3–C2–N7	115	115	115	115	115	116
C2–N7–C8	121	121	121	121	121	120

**Table 2:** Binding energies (Δ*E*_B1_, kJ/mol) of DCD and different functional monomers in different ratios at the M062X/6-31G(d,p) level.

RatioComplexes	DCD-AM	DCD-MAA	DCD-MBA	DCD-IA
1:1	10.33	73.60	0.05	3.28
1:2	−40.03	−59.09	−55.06	−42.148
1:3	−93.73	−106.41	−114.47	−120.14
1:4	−122.02	−143.47	/	−134.45
1:5	−151.75	−191.40	/	/

**Table 3:** Binding energies (Δ*E*_B2_) between DCD as well as MAA and different cross-linkers at the M062X/6-31G(d,p) level (kJ/mol).

Species	EGDMA	DVB	PETA	TRIM
DCD	−36.49	−34.92	−33.61	−53.30
MAA	−23.63	−16.02	−43.58	−37.54

## Abbreviations

MIPs	Molecularly Imprinted Polymers
DCD	Dicyandiamide
MAA	Methacrylic Acid
PETA	Pentaerythritol Triacrylater
AM	Acrylamide
MBA	*N*,*N*’-methylenebisacrylamide
IA	Itaconic Acid
DVB	Divinylbenzene
EGDMA	Ethylene Glycol Dimethacrylate
TRIM	Trimethylolpropane Trimethylacrylate
MEPs	Molecular Electrostatic Potentials
NIPs	Non-imprinted Polymers
SEM	Scanning Electron Microscope
MAM	Melamine
CYR	Cyromazine
TRI	Trithiocyanuric
BSSE	Basis Set Superposition Error
